# Effect of Cowpea and Pumpkin Powders on the Physicofunctional Properties, Total Phenolic Content, Antioxidant Activity, and Consumer Acceptability of Soup

**DOI:** 10.1155/2024/3596783

**Published:** 2024-08-06

**Authors:** Nyarai Mungofa, Daniso Beswa

**Affiliations:** ^1^ Department of Life and Consumer Sciences College of Agriculture and Environmental Sciences University of South Africa, Science Campus, Johannesburg 1709, South Africa; ^2^ Department of Biotechnology and Food Technology Faculty of Science University of Johannesburg Centre for Innovative Food Research (CIFR), Doornfontein Campus, Johannesburg 2028, South Africa

**Keywords:** antioxidant activity, consumer acceptability, functional properties, physical properties, soup formulations, total phenolic content

## Abstract

Cowpea (*Vigna unguiculata*) and pumpkin (*Cucurbita maxima*) play a pivotal role as affordable, nutritious food sources for humans. Low-income households can significantly benefit from supplementing their diet with nutritious and cost-effective locally available ingredients. The aim of this research was to develop a cost-effective soup formulation using ingredients that are readily available from a household garden and suitable for use in the kitchens of families with limited financial resources. The effect of cowpea and pumpkin powders on physicofunctional properties, total phenolic content (TPC), antioxidant activity (AA), and consumer acceptability of the soup were determined. Three composite soup mixes were prepared using various parts of cowpea and pumpkin at a ratio of 1:1. A control soup sample was developed, and the experimental soups were prepared by supplementing the control soup with 5%, 10%, or 15% of each composite soup mix, respectively. The physical properties, functional properties, TPC, AA, and consumer acceptability of soup were determined. The control soup had an appealing golden brown colour. Formulations 1 and 3 showed the highest relative viscosity (80.67–88.91 cP). All the experimental soup formulations had higher rehydration ratios (8–14.7 g/g) and water absorption capacities of 185.7–263.3 g/g compared to the control at 7.7 g/g and 65.7 g/g, respectively. The TPC of Formulation 2 (F2) (0.32–0.54 mg of gallic acid equivalent (GAE)/100 g powder) and Formulation 3 (F3) (0.54–0.63 mg GAE/100 g powder) was higher than Formulation 1 (F1) (0.25–0.32 mg GAE/100 g powder) and the control (0.44 mg GAE/100 g powder). Overall, the cowpea seed powder plus pumpkin fruit powder added at 10% in F2 appeared nearly optimal as a soup mix. It produced an attractive golden brown soup with increased swelling power (SP) and viscosity to assist in swallowing. Soup F1 and F2 scored high in appearance (7.27 and 7.0), aroma (7.1 and 6.7), taste (6.6 and 6.3), and overall acceptability (6.5 and 6.4). Despite having TPC and AA lower compared to F3, F2 containing 15% cowpea seed powder and pumpkin fruit has the potential to be further developed and commercialised due to the relatively high overall consumer acceptability and high acceptability in all sensory attributes.

## 1. Introduction

Soups are widely regarded as one of the most popular culinary offerings across the globe, with a rich history spanning centuries. This cuisine can be prepared using a diverse array of ingredients, including vegetable fruits, tubers, bulbs, leafy vegetables, and legumes [1] which significantly contribute towards the nutritional value of soups [2]. In addition to these plant-based components, there are also lesser-known plant-based ingredients that can significantly enhance the nutritional value and health-promoting benefits of the soup. Examples of such ingredients include cowpea (*Vigna unguiculata*) and pumpkin (*Cucurbita maxima*). Cowpea, a legume crop of significant nutritional value, is native to Africa [3], while pumpkin, a fruit that resembles a gourd, is indigenous to both tropical and subtropical regions and is widely recognised for its exceptional nutritional composition [4].

In South Africa, cowpea and pumpkin are cultivated and consumed primarily in the Limpopo Province [5–9]. Although the leaves of cowpea and pumpkin plants are commonly used, all parts of these plants are edible. Cowpea, for instance, produces tender green leaves, immature green pods, and nearly mature fresh-shelled grains that are highly nutritious [10–12]. Cowpea leaves possess a rich nutritional profile, serving as a crucial reservoir of essential nutrients such as proteins, vitamins, and minerals [13, 14]. Seke et al. [15] have reported that cowpea leaves possess significant levels of carotenoids, including lutein, violaxanthin, *α*-carotene, 9-*cis*-*β*-carotene, and zeaxanthin. Abebe and Alemayehu [13] and Sombié et al. [16] have found that cowpea leaves contain a considerable amount of phytochemicals such as flavonoids and phenolic acids. These leaves are also characterized by their low-fat content and significant contribution to dietary fibre intake [17].

Dry, mature cowpea seeds can also be processed into flour and used to make various foods, including cakes, bread, and couscous, as well as complementary food ingredients [18, 19]. According to Abebe and Alemayehu [13], the seeds provide a valuable supply of carbohydrates and protein, while Tzanova et al. [20] found that they also contain a significant amount of unsaturated fatty acids. The seed coat of cowpea is primarily where the polyphenolic compounds are concentrated, as highlighted by Tzanova et al. [20] and Adjei-Fremah, Jackai, and Worku [21]. Similarly, pumpkins offer nutritious leaves and flowers that can be cooked and consumed as vegetables, while pumpkin fruit contains seeds that can be cooked, roasted, or milled into powder and incorporated into various food formulations [18, 22, 23]. Pumpkin leaves have been found to contain significant amounts of vitamin A, vitamin C, calcium, iron, and potassium, as well as bioactive compounds that exhibit high levels of activity, according to recent studies [24–29]. Similarly, pumpkin flowers are rich in minerals, polyunsaturated fatty acids, and antioxidants, as reported by Santos et al. [30]. Moreover, both fresh pumpkin leaves and flowers are known to contain important bioactive compounds that possess pharmacological properties [31]. Pumpkin seeds have been recognised as an excellent source of protein; phenolic compounds; trace elements such as copper, iron, zinc, and manganese; and unsaturated fatty acids such as palmitic (C 16:0), stearic (C 18:0), oleic (C 18:1), and linoleic (C 18:2) [32, 33]. On the other hand, the fruit itself is abundant in carotenoids (*β*-carotene, lutein, and violaxanthin) and essential nutrients like potassium, iron, manganese, magnesium, phosphorus, vitamin C, vitamin E, and phytosterols [34–36]. From a nutritional standpoint, both cowpeas and pumpkins possess elevated levels of essential nutrients [35–38].

The significant nutritional value of these two food items suggests their potential in the development of nutrient-dense foods, such as soups, which can be utilized to complement starchy meals such as maize meal porridge. It is worth noting that white maize meal porridge is a widely consumed food in South Africa, with an average daily consumption of 460 g of maize per person in the Eastern Cape Province [39]. However, it was noted that this staple food is deficient in certain essential nutrients [40–45]. A decrease in the consumption of these vital nutrients may result in health-related conditions. Cowpea has a significantly higher protein content compared to cereals, with 2–4-fold more protein [13, 46], and is rich in lysine and tryptophan [47]. On the other hand, pumpkins are known for their high levels of provitamin A carotenoids, surpassing maize which lacks vitamin A [48–51].

An extensive examination of the existing literature has uncovered a lack of published studies describing the development of food products utilizing various parts of cowpea and pumpkin as complementary sources of nutritious constituents. During the investigation into the viability of incorporating various parts of cowpea and pumpkin as complementary food components, multiple factors were examined, including the physical and functional characteristics, total phenolic content (TPC), antioxidant activity (AA), and consumer acceptability of pumpkin and cowpea, to assess the appropriateness of integrating cowpea and pumpkin into soup formulations.

## 2. Materials and Methods

### 2.1. Materials and Sample Preparation

#### 2.1.1. Materials

The vegetables used in this study were planted in Burgersfort, Limpopo Province, South Africa, and harvested manually during the 2019 and 2020 cropping seasons. Raw soup materials such as dry carrot granules, potato powder, onion powder, mushroom powder, salt, brown sugar, and tomato powder were procured locally from Burgersfort supermarkets (Pick n Pay, Shoprite, and Karachi Corner). The Unidri 20® powdered maltodextrin was sourced locally (Unidri®, Tongaat Hulett Starch, Germiston, South Africa). Laboratory chemicals were provided by the Food Science Laboratory, University of South Africa (UNISA), Florida Campus, South Africa. These were purchased from Sigma-Aldrich (Modderfontein, Johannesburg, South Africa).

#### 2.1.2. Sample Preparation

The cowpea and pumpkin leaves were manually harvested when they reached the mature green stage, and the pumpkin flowers were collected when they were still tender. The cowpea seeds, pumpkin fruit, and pumpkin seeds were harvested only when they had fully ripened.

##### 2.1.2.1. Pumpkin Flowers

Briefly, pumpkin flowers were sorted before being washed and blanched. Blanching involved fully submerging the whole flower in boiled water for 2 min. The flowers were drained, cooled, and oven-dried at 60°C until completely dry. Then, they were milled and screened through a clean mesh sieve with 2 mm spaces before being packed in low-density polyethylene bags and stored at 4°C in a refrigerator until use.

##### 2.1.2.2. Pumpkin Fruit

The preparation of the pumpkin fruit was described by Różyło et al. [52] and Pongjanta et al. [53]. Briefly, the fruit was cleaned and peeled, the seeds were removed, and the flesh was cut into 2 cm^3^ pieces. These pieces were soaked in 0.1% (*w*/*v*) citric acid for 15 min to prevent oxidation before being drained, crushed, and pulped. The pulp (flesh residue) was screened through a 0.35 mm sieve and spread on stainless steel trays before being oven-dried at 65°C for 8 h. The dried pulp was milled and screened using a 2 mm mesh sieve before being packaged in polyethylene bags and stored in a refrigerator at 4°C.

##### 2.1.2.3. Cowpea Leaves

The preparation of the leaves was conducted as described by Idris [54]. Briefly, this involved sorting the cowpea leaves before they were washed and blanched by fully submerging the flowers in a pot of boiling water for 2 min. They were drained, cooled, and oven-dried at 60°C until they reached a constant weight. The dry leaves were then milled, screened (2 mm mesh sieve), packed in polyethylene bags, and stored at 4°C until use.

##### 2.1.2.4. Cowpea Seeds

Cowpea seeds were sorted, cleaned, and soaked in tap water (1:10 *v*/*v*) at 25°C for 24 h. They were then drained, dehulled, and boiled in fresh tap water for 30 min. The boiled seeds were cooled and oven-dried at 130°C for 6 h. The dried seeds were milled, screened (2 mm mesh sieve), packed in polyethylene bags, and stored in a refrigerator at 4°C until use.

#### 2.1.3. Recipe Development and Preparation of Soup Mixtures

The process of developing the recipe involved preparing three different composite mixes using various edible parts of cowpea and pumpkin. The first mix (mix A) consisted of cowpea leaf powder and pumpkin seed powder in equal proportions. Mix B, the second composite mix, was made up of cowpea seed powder and pumpkin fruit powder also in equal proportions. The last (mix C) contained cowpea leaf powder and pumpkin flower powder also in equal amounts. A control soup recipe was prepared, which comprised dried carrots, potato powder, onion powder, mushroom powder, powdered maltodextrin, hydrogenated fat, salt, brown sugar, and tomato powder (Table 1). The composite mixes were not added to the control soup mix. To develop the experimental soup formulations, three variations (Formulation 1 [F1], Formulation 2 [F2], and Formulation 3 [F3]) were prepared using the control soup recipe as the base, with the composite mixes incorporated at 5%, 10%, and 15%, respectively (Table 1). F1 was divided into three different variations. The first variation consisted of a mixture of 5% cowpea leaf powder and pumpkin seed powder. The second variation contained 10% cowpea leaf powder and pumpkin seed powder, while the third variation contained 15% cowpea leaf powder and pumpkin seed powder. For F2, the first variation included a mix of 5% cowpea seed powder and pumpkin fruit powder. The second variation had 10% cowpea seed powder and pumpkin fruit powder, and the third variation contained 15% cowpea seed powder and pumpkin fruit powder. The first variation of F3 is composed of 5% cowpea leaf powder and pumpkin flower powder mix. The second variation contained 10% cowpea leaf powder and pumpkin flower powder mix, and the third variation consisted of 15% cowpea leaf powder and pumpkin flower powder mix. The composite mixes were packed in polyethylene bags and stored at 4°C.

### 2.2. Physical Properties of Soup and Soup Mixes

#### 2.2.1. Colour Measurement of Soup and Soup Mixes

A precalibrated Konica Minolta Chroma Meter CR-400 (Osaka, Japan) was used to measure the sample colour. The readings were recorded as *L*^∗^, *a*^∗^, and *b*^∗^ and chroma values, whereby CIE *L*^∗^ (100 = white; 0 = black) indicated lightness; CIE *a*^∗^ measures chromaticity (i.e., the quality of colour), with negative values showing greenness and positive values indicating redness; and CIE *b*^∗^ measures chromaticity, with negative values indicating blueness and positive values showing yellowness, and chroma (*C*^∗^) (colour saturation, increasing from 0).

#### 2.2.2. Viscosity of Soup and Soup Mixes

Exactly 10 g of each different soup composition was weighed and dispersed in 100 mL of distilled water. The mixture was stirred with heating until it reached boiling point, started thickening, and then continued to boil for a further 5–10 min with constant stirring. However, it was noticeable that once stirring ceased, the mixture had a nonuniform composition. Soups were then cooled to room temperature before analysis was started. The measurements were performed on the Brookfield Ametek RST Rheometer (type: RSTSST, S/N: 7123080) with a CCT-25 spindle (S/N: 0400174). The software used was Rheo3000, v2.0. The method parameters are as follows: time: 60 s; speed: 0–1300 rpm; reading points: 60; no temperature control.

### 2.3. Functional Properties of Soup and Soup Mixes

#### 2.3.1. Water Absorption Capacity (WAC)

A slightly modified method by Olawoye and Gbadamosi [55] was used to determine the WAC of the soup mixes. Briefly, 15 mL of distilled water was added to 1 g of the formulated soup mix in a preweighed centrifuge tube. The tube and its content were agitated on an IKA orbital shaker (KS 130 Basic Shaker, Lasec laboratories) for 2 min and then centrifuged (Hermle Laboratory Centrifuge, Lasec, South Africa) for 20 min at 4000 rpm. The tube and its content were agitated on an IKA orbital shaker (KS 130 Basic Shaker, Lasec laboratories) for 2 min and then centrifuged for 20 min at 4000 rpm. The clear supernatant was decanted, and the mass of the sediment in each centrifuge tube was measured. The quantity of water bound by the sample was determined by subtracting the mass of the centrifuge tube plus the mass of the soup mix from the mass of the tube plus the soup mix sediment. Using the equation below, the difference between these masses was then calculated and the WAC was expressed as a gram of water absorbed per gram of soup mix [56].

 $$\begin{array}{c} \text{WAC}\left(\text{g}/\text{g}\right)=\frac{\text{W}3-W2}{W1} \end{array}$$

where *W*_3_ is the weight of the empty tube + the sample after centrifuged and decanted, *W*_2_ is the weight of empty tube + sample before centrifuging, and *W*_1_ is the weight of a sample.

#### 2.3.2. Swelling Power (SP)

The method by Adegunwa et al. [57] was used to determine the SP of the soup mixes. Briefly, 1 g of soup powder was weighed into each of four preweighed 50-mL centrifuge tubes and gently mixed with 50 mL of distilled water. Respective soup slurries were then heated in a water bath at 70°C, 80°C, 90°C, and 100°C, respectively, for 15 min. To prevent the soup from clumping, each slurry was gently stirred during heating. After 15 min, the tube with the soup slurry was centrifuged at 3000 rpm for 10 min using a centrifuge (Hermle Laboratory Centrifuge, Lasec, South Africa). Each supernatant was decanted promptly after centrifuging, and the weight of the sediment was measured and recorded. The content of moisture in each sediment was determined relative to the dry matter as follows:

 $$\begin{array}{c} \text{Swelling power}=\frac{\text{weight of wet mass sediment }\left(\text{g}\right)}{\text{weight of dry matter in the gel }\left(\text{g}\right)}\times 100 \end{array}$$

#### 2.3.3. Dispersibility

Dispersibility was determined using the method described by K. Kulkarni, D. Kulkarni, and Ingle [58]. The percentage dispersibility was determined by introducing 10 g of the sample into a stoppered measuring cylinder with a capacity of 100 mL. Distilled water was then added to achieve a total volume of 100 mL. The mixture was vigorously stirred for 5 min and left to settle for 3 h. Dispersibility was calculated by subtracting the volume of deposited particles recorded at a time *t* (*V*_*t*_) from the total volume of particles just after stirring (*V*_0_) and dividing by the total volume of particles just after stirring and multiplying by 100 [59].

 $$\begin{array}{c} \text{Dispersibility }\left(\%\right)=\frac{V_{0}-V_{t}}{V_{0}}\times 100 \end{array}$$

where *V*_0_ is the total volume of particles just after stirring and *V*_*t*_ is the volume of deposited particles recorded at a time *t*.

#### 2.3.4. Rehydration Ratio

The rehydration ratio provides details of the mass of rehydrated and drained food relative to the mass of the original material [60]. This measurement was performed as described by Hanan et al. [61]. Exactly 2 g of each of the formulated cowpea–pumpkin soup powders was mixed with 20 mL of distilled water in a glass beaker and immersed in water baths set at 40°C, 60°C, or 80°C, respectively, while being agitated at a constant speed of 100 rpm. After 10 min, the samples were removed from the water baths, blot-dried using tissue paper (to remove the extra solution), and weighed. The rehydration ratio is the ratio of the weight of rehydrated samples relative to the dry weight of the sample, as shown by the following equation:

 $$\begin{array}{c} \text{Rehydration ratio }\left(\text{g}/\text{g}\right)=\frac{\text{weight of the sample at }t\;\min\text{ of rehydration}}{\text{dry weight of the sample before hydration}} \end{array}$$

### 2.4. TPC and AA of Soup Mixes

#### 2.4.1. Preparation of Phenolic Extracts

The phenolic compounds from each cowpea and pumpkin soup powder sample were extracted using the method described by Mamphiswana, Mashela, and Mdee [62]. Each sample was extracted with 30 mL of 80% aqueous methanol containing 1% HCl in three phases as follows: 10 mL of solvent was added to 0.3 g of the sample in a conical flask that was then completely covered with aluminium foil. Each sample was vortex mixed (IKA, Staufen, Germany) at 10 min intervals for 2 h, transferred to 40-mL plastic centrifuge tubes, centrifuged at 3500 rpm for 10 min (25°C), and decanted, retaining the supernatant. Each sample was vortex mixed (IKA, Staufen, Germany) at 10-min intervals for 2 h, transferred to 40 mL plastic centrifuge tubes, centrifuged (Hermle Laboratory Centrifuge, Lasec, South Africa) at 3500 rpm for 10 min (25°C), and decanted, retaining the supernatant. The sample residue was rinsed again with 10 mL of the solvent, vortex mixed (IKA, Staufen, Germany) for 20 min before being centrifuged again as above, and decanted, keeping the supernatant. This step was repeated as described. The supernatants were combined, stored in a glass bottle covered with aluminium foil, and kept in a cold room until analysed.

#### 2.4.2. Determination of TPC

The Folin–Ciocalteu method described by Hussain et al. [63, 64] and Hussain et al. [26–29] with slight modifications was used to determine the TPC of cowpea and pumpkin soup mixes. Gallic acid was used as the standard for this assay. To perform the assay, 0.5 mL of extracts were mixed with 10 mL of distilled water in a volumetric flask. Then, 2.5 mL of Folin–Ciocalteu reagent was added to the flask, followed by shaking and incubating in the dark. After 2 min, 7.5 mL of sodium carbonate solution (20 g/100 mL) was added to each flask. The contents were mixed and made up to volume with deionized water. The flask was stoppered and thoroughly mixed by inverting it several times. Following the addition of sodium carbonate, the flask was allowed to stand for 2 h before measuring the absorbance at 760 nm using a UV/Vis spectrophotometer (VWR UV-1600 PC, Radnor, PA, United States). A reagent blank was also prepared using distilled water. The concentration of total phenolic compounds in the extract was expressed as milligrams of gallic acid equivalent (GAE) per 100 g sample (mg GAE/100 g). All samples were analysed in triplicate.

#### 2.4.3. Determination of AA

##### 2.4.3.1. DPPH Radical Scavenging Activity (RSA)

The scavenging ability of soup mix antioxidants against the stable radical DPPH was measured using a method described by Kaur et al. [65] and Anggraini et al. [66]. To conduct the assay, a 3.9-mL aliquot of a 0.0634 mM DPPH solution in 95% methanol is combined with 0.1 mL of each extract and vigorously shaken using a vortex mixer (IKA, Staufen, Germany). Then, a change in absorbance of the soup mix extracts was measured at 515 nm over a period of 30 min. The DPPH RSA was calculated as follows:

 $$\begin{array}{c} \text{RSA }\left(\%\right)=100\times \frac{\left(A_{0}-A_{1}\right)}{A_{0}} \end{array}$$

where *A*_0_ is the beginning absorbance at 515 nm, obtained by measuring the same volume of solvent, and *A*_1_ is the final absorbance of the sample extract at 515 nm. Methanol (95%) was used as a blank. The IC_50_ was calculated from the graph plotting the scavenging percentage against test sample concentration (micrograms per milliliter).

##### 2.4.3.2. ABTS RSA

The total antioxidant potency of the soup mix extract was evaluated using the ABTS^•+^ radical decolourisation assay, as detailed by Murugan et al. [67]. The process involved the preparation of the ABTS^•+^ solution by reacting a 7 mM ABTS aqueous solution with 2.4 mM potassium persulfate in the dark at room temperature for a maximum of 16 h. Before the assay, the ABTS^•+^ solution was dissolved in ethanol at a ratio of approximately 1:89 (*v*/*v*) and allowed to reach equilibrium at 25°C. For the test solution, 1 mL of the diluted ABTS solution was mixed with about 30 *μ*L of soup mix extract and 10 *μ*L of Trolox (with a final concentration ranging from 0 to 15 *μ*M) in ethanol. The negative control consisted of 1 mL of the diluted ABTS solution and 30 *μ*L of ethanol. After incubation, the absorbance of the soup mix extract and the standards (BHT and rutin) was measured against the ethanol blank at 734 nm. The concentration of Trolox that exhibited equivalent AA was expressed as micromolars per gram of soup mix extract (micromolar TE/g sample).

### 2.5. Ethical Considerations

The research work was conducted in the UNISA laboratories in Florida under the supervision of Dr. Daniso Beswa. The study received ethics approval from UNISA Ethics, with the reference number 2019/CAES/076. To ensure the safety of students, staff members, and the environment, the laboratory code of conduct and safety rules were strictly adhered to, preventing any potential harm or injuries.

### 2.6. Consumer Acceptability of Soup

#### 2.6.1. Preparation of Soup Samples

The soup was prepared by combining one part of cowpea–pumpkin composite formulations with three parts of boiling water (Table 1). A control soup (with 0 g of composite mixes) was also prepared. After the ingredients were mixed, the mixture was allowed to boil on a stove at 94°C for 5 min while continuously stirring to ensure complete dissolution of the powders. Then, the soup was removed, cooled, and allowed to cool to 25°C. The soup samples were labelled with three-digit random numbers and presented to the sensory panel in a randomised order as described by Byarugaba, Nabubuya, and Muyonga [68]; Granato, Masson, and Ribeiro [69]; and Netshishivhe et al. [70]. The soup samples were carefully sealed with cling film and stored at room temperature (25°C) for an estimated duration of 1 h, allowing ample time to prepare the sensory panel for the evaluation process.

#### 2.6.2. Evaluation of Soup Samples

Due to the COVD-19 pandemic and lockdown restrictions, the sensory evaluation was conducted outside the laboratory in a noise- and odour-free venue. Approval was obtained from the municipal manager to perform the consumer acceptance test in Fetakgomo Tubatse Local Municipality, Limpopo Province, South Africa. To evaluate the various vegetable soups, a panel of 70 (*n* = 70) untrained consumers who were over the age of 18 years were randomly selected based on their availability as determined during their recruitment. The panellists were all selected from rural communities, representing the main beneficiaries of the results and recommendations from this study. The panelists gave their consent by signing an informed consent form, thereby agreeing to take part in the study and confirming their understanding of the study aim. To comply with COVID-19 rules and regulations, social distancing was observed by seating the panellists 1 m apart as well as to prevent them from influencing each other and no talking was allowed during the evaluation process.

The consumer panel comprised males (*n* = 29) and females (*n* = 41) who confirmed that they were not allergic to the ingredients used to formulate the soup recipe. The panel evaluated a spoonful (25–30 mL) of hot soup samples (in transparent plastic cups) for visual appearance, odour, mouth/texture, taste, and overall acceptability using a 9-point hedonic scale (9 = *liked very extremely*, 8 = *liked very much*, 7 = *liked moderately*, 6 = *liked slightly*, 5 = *neither liked nor disliked*, 4 = *disliked slightly*, 3 = *disliked moderately*, 2 = *disliked very much*, and 1 = *disliked extremely*) [71]. The panel was provided with tap water in separate containers to rinse their palates before and between tastings. The panellists wrote their responses directly onto a questionnaire which was provided to each panellist.

### 2.7. Statistical Analysis

The statistical analysis was conducted utilizing the Statistical Package for the Social Sciences (SPSS) Version 28.0 (IBM, New York, United States). A one-way analysis of variance (ANOVA) was used to evaluate significant differences (*p* < 0.05) between the means. The outcomes were presented as the mean values ± standard deviation of three replicates, and the mean comparison was performed using Duncan's multiple range test and Pearson's correlation and was conducted using 2023 XLStat software (Addinsoft, Paris, France) [72].

## 3. Results and Discussion

### 3.1. Effect of Cowpea Powder and Pumpkin Powder on the Physical Properties of Cowpea–Pumpkin Composite Soup

#### 3.1.1. Appearance and Colour of Cowpea–Pumpkin Composite Soup Formulations

**Figure 1:**
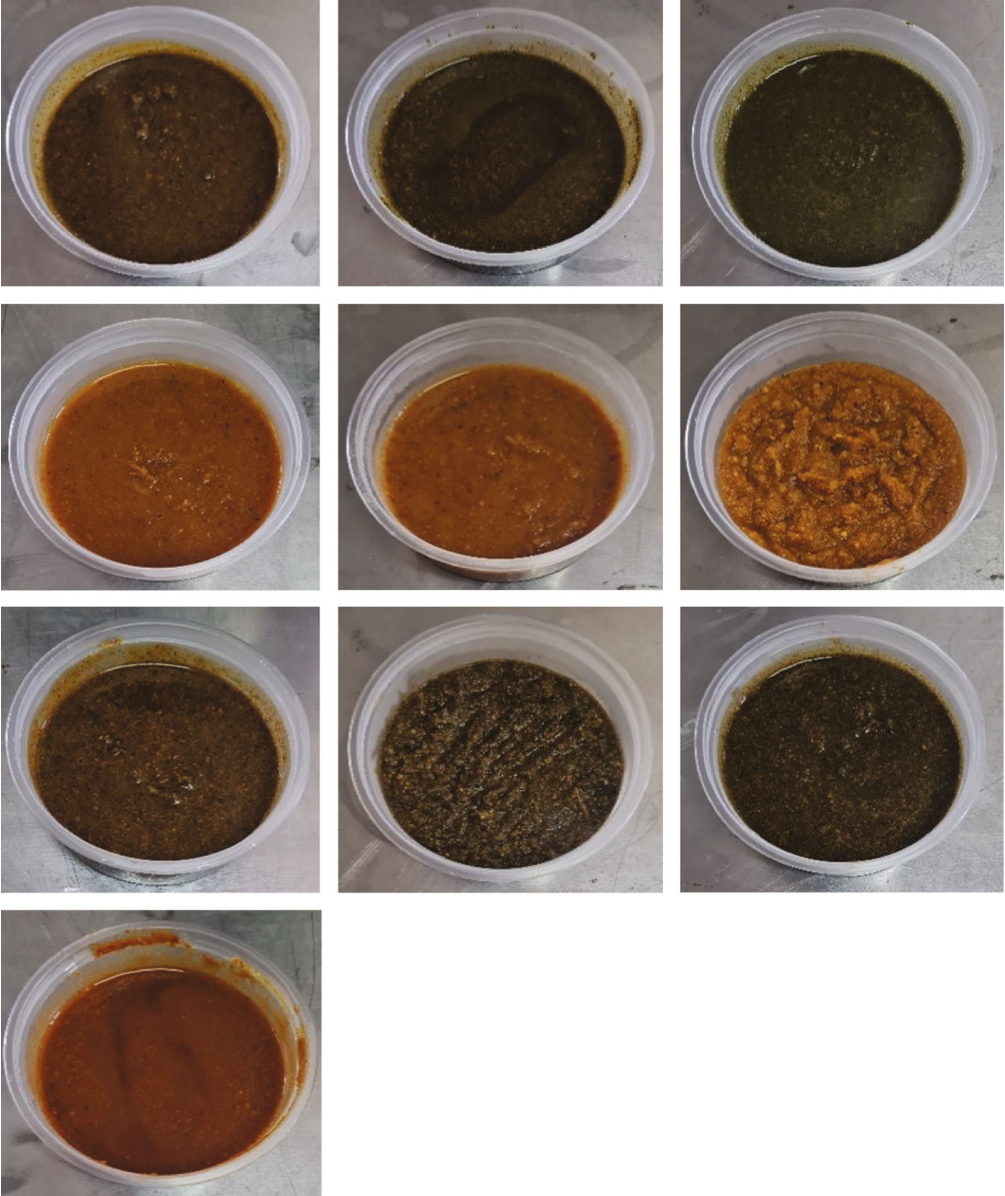
Photographs showing variations in the colour of cowpea–pumpkin composite soup formulations (photographs taken by N.M.).

Photographs of cowpea–pumpkin composite soups are shown in Figure 1. The control soup mix samples had an appealing golden brown colour. Regarding F1, following the addition of 5% of the composite mix, the colour of the soup deepened slightly with slight gold and green shades which lacked consumer appeal when compared to the control. As the cowpea–pumpkin composite mixes increased to 10% and then 15%, the green colour, in particular, increased in intensity. From a colour perspective, the appearance of F1 was similar to that of F3. F3 exhibits a colour that has deviated slightly from an intense green hue towards a more brownish tone, a change that is evident through visual observation and further confirmed by the positive *a*^∗^ and *b*^∗^ values. This particular shade may not be one that consumers typically associate with soups; thus, it could be deemed unappealing or unsatisfactory in the eyes of the consumers. Cowpea leaf powder was common to these two soup formulations and so contained chlorophyll pigments [73] that imparted a dark green colour to the soup, and the intensity of the green colour increased as the concentration of composite mixes increased in the formulations to 10% and then 15%.

In terms of texture, F1 was different from F3 in appearance. F3 was relatively thicker and grainier. In addition to its dark colour, F3 had a coarse texture. Cowpea leaf and pumpkin flower powder can also make soup appear thicker and grainier due to their high fibre content which adds bulk to the soup. The fibrous nature of these ingredients also contributed to the grainier texture as they do not dissolve completely. In contrast, F2 with a 5% composite mix was an eye-catching, light golden brown colour which was similar to the control soup. The difference between the appearance of F2 soups and those of F1 and F3 was the exclusion of vegetable leaf powders from the composite mix, and this enhanced the consumer appeal of F2. While all the F2 soups retained the colour of the control soup, the texture of the 10% and 15% F2 soup mixes increased in graininess and appeared less appealing. In brief, a visual comparison of the three soup formulations according to their percentage composition indicated that F2 soup colour and texture were most appealing at 5% and 10% powdered cowpea seed and pumpkin fruit.

The colour of food largely affects its consumer appeal which ultimately has an influence on consumer expectations and purchase decisions [74]. A colorimetric comparison of the control soup and F2 soup samples showed that the intense golden brown colour of these soups was associated with relatively high chroma values which ranged from 17.02 for the control soup and from 17.42 to 23.84 for the three F2 soups containing increasing amounts of powdered cowpea seed and pumpkin fruit (Table 2). The lightness intensity of the golden brown colour increased with the addition of mixes from 5% through 10% and on to 15% composite mixes, as indicated by the high *L*^∗^ mean values and chromaticity values of F2 compared to the *L*^∗^ value and chromaticity value of the control soup. This observation was also supported by the high positive *b*^∗^ values of F2 when compared to the control soup. High positive *b*^∗^ values (responsible for yellowing) were probably due to the release of anthocyanins which are relatively high in the pumpkin fruit. The golden brown colour of F2 soup samples was probably imparted by carotenoids which are present in pumpkin fruit and flours [32, 35, 36, 76]. Carotenoids are abundantly occurring natural pigments responsible for the yellow, orange, and red colour of many fruits, vegetables, algae, and bacteria [78, 79].

The addition of more cowpea leaves resulted in a darker colour of the soups F1 and F3, as shown by a decrease in *L*^∗^ values, while *a*^∗^ values shifted from positive (redness) to negative (greenness) for F1. In these formulations, the soup samples with a 5% composite mix had the highest value of lightness (*L*^∗^). The intensity of the green colour of the soup samples from F1 and F3 could be attributed to the presence of cowpea leaves. Green plant leaves are known to contain a high content of chlorophyll, a pigment responsible for the green colour [80]. Increasing the concentration of the composite mix resulted in a further decrease in *L*^∗^ values (74.06–60.92 for F1, 72.49–55.29 for F2, and 71.37–52.63 for F3, respectively), and *b*^∗^ values (+ve) and *a*^∗^ values (+ve) also decreased. The darker colour of the soup samples blended redness and yellowness. However, the overall colour difference indicates a significant effect of cowpea leaf addition on the colour of soups F1 and F3. These formulations (F1 and F3) were visually different from the control soup due to the saturated green colour and therefore may not appeal to consumers who are accustomed to soups with colours ranging from brown to golden brown.

#### 3.1.2. Rheological Properties of Cowpea–Pumpkin Composite Soup Formulations

Viscosity is one of the important attributes of liquid foods such as soups and has an effect on the mouthfeel and swallowability of food [81, 82]. The selected rheological properties of cowpea–pumpkin soup samples measured in this study are presented in Table 3. The viscosity of the control soup (33.21 cP) was significantly (*p* < 0.05) higher than that of F1 and F2 with a 5% composite mix (21.09 and 24.13 cP, respectively). However, the viscosity of formulations with 5% composite mix, F2 with 10% composite mix, and F3 with 10% and 15% composite mix were more than twofold higher than that of the control soup. The control soup exhibited a shear stress of 13.92 Pa at a shear rate of 497.23 s^−1^. Among the F1 variants of cowpea leaf powder and pumpkin seed powder, the variation with 5% was more viscous, as shown by the significantly (*p* < 0.05) higher viscosity mean value of 88.91 cP with a shear stress of 21.2 Pa at a shear rate of 497.2 s^−1^. This was followed by the variations with the highest concentration of cowpea leaf powder and pumpkin seed powder (15%) at a viscosity of 56.6 cP and shear stress of 15.1 Pa at a shear rate of 497.2 s^−1^. These values show that the addition of cowpea leaf powder and pumpkin seed powder in F1 significantly (*p* < 0.05) increased its viscosity with reference to the control soup at the same shear rate. The increase in viscosity of F1 was probably due to the presence of dietary fibres in pumpkin seed and cowpea leaves which have the ability to bind water, tending to increase the consistency of the products and so leading to the formulation of viscous gels and consequently promoting higher viscosities [13, 83]. This phenomenon could also be attributed to the molecular interactions occurring within the samples, hindering their ability to rehydrate and form crosslinks due to the high concentration of fibre which competes for water [84]. The F2 soup sample with 10% cowpea seed powder and pumpkin fruit powder was the thickest among the variations of F2. This sample had a viscosity of 62.75 cP, a shear stress of 38.5 Pa, and a shear rate of 653.2 s^−1^. In contrast, the 5% F2 variation exhibited the lowest viscosity (21.1 cP) and shear stress (7.8 Pa) among all the formulations in this study, including the control soup. According to Santamaria et al. [85], viscosity is directly related to the amount of starch present in the food and the reduction in viscosity of F2 (5%) could have been caused by the lower starch content in the lowest concentrations of cowpea seed powder and pumpkin fruit powder (5%).

The incorporation of 10% cowpea leaf powder and pumpkin flower powder in F3 resulted in soup samples with a significantly (*p* < 0.05) higher viscosity of 80.7 cP, a shear stress of 65.1 Pa, and the highest shear rate of 823.8 s^−1^ among the variations of F3. The lowest viscosity, at 5% addition to the composite mix, was also observed in F2. The inclusion of cowpea in the formulation could have potentially impacted the viscosity, possibly because of the interaction between starches and proteins. This interaction has the potential to influence the gelatinization and retrogradation processes of the starches, leading to changes in viscosity [86]. Presumably, with a 5% composite mix, the inherent enzymes like proteases found in cowpea leaf and pumpkin flower [87] disintegrated the proteins, leading to a decline in the protein content of the soup and consequently lowering its viscosity. Conversely, a notable rise in viscosity noted upon incorporating 10% of the composite mix might be attributed to the increased protein concentration from the mixture and the higher plant material concentration possibly introducing starches and fibre, which could further boost the soup's viscosity.

The variation in viscosity and shear is not linear as the formulations increase from 5% through 10% to 15%. Among the F1 variations (cowpea leaf powder and pumpkin seed powder), it was noted that as the viscosity increased, there was a decrease in shear stress and shear rate, and vice versa, characteristic of non-Newtonian foods [88]. In contrast, F2 variations (cowpea seeds and pumpkin fruit) and F3 variations (cowpea leaf powder and pumpkin flower) showed an increase in shear stress and shear rate as the viscosity increased. This means that an increase in shear rate and shear stress increased molecular alignment in the soup and the resistance of the soup to flow, resulting in an increase in viscosity. This could be due to the addition of pumpkin seed powder in F1 and pumpkin fruit and pumpkin flower in F2 and F3. F2 and F3 could be examples of Newtonian food, foods with a constant viscosity [88].

It can be concluded that F1, consisting of cowpea leaf and pumpkin seed powder at a concentration of 5%, and F3, containing cowpea leaf powder and pumpkin flower at concentrations of 10% and 15%, exhibited the desired viscosity. Consequently, soups with higher viscosity are considered to be thicker and of superior quality [89]. When consuming food that contains both liquid and solid components, the liquid portion tends to enter the hypopharynx before swallowing, resulting in a heightened risk of aspiration [90]. Individuals with chewing and swallowing disorders, such as dysphagia, may encounter difficulties in ingesting regular food and liquids, necessitating a modified texture diet [91]. Therefore, soups with higher viscosity, as observed in this study, would facilitate easier swallowing and could be recommended for children, the elderly, and individuals with dysphagia, enabling them to maintain adequate nutrition. Therefore, when developing a liquid food product, it is important to ensure that its viscosity suits the swallowing abilities and limitations of the target consumers. Low-viscosity soups with porous and unstable consistencies pose a higher risk of entering the airways during swallowing. Soups are generally perceived as easier to swallow and could be recommended for individuals with swallowing difficulties, including young children.

### 3.2. Effect of Cowpea Powder and Pumpkin Powder on the Functional Properties of Cowpea–Pumpkin Composite Soup

The functional properties of a soup refer to how food constituents within the ingredients behave and interact with each other during processing. Their molecular interaction has an effect on how the final product looks, feels, and tastes [92]. These properties include WAC, SP, dispersibility, solubility index, foaming capacity, foam stability, and rehydration ratio. However, this study only focused on WAC, SP, dispersibility, and rehydration ratio. The results for the studied functional properties are discussed in subsequent sections (Sections 3.2.1–3.2.4).

#### 3.2.1. WAC of Cowpea–Pumpkin Soup Mixes

The functional properties of cowpea–pumpkin composite soup are presented in Table 4. The WAC, also called water holding capacity, is the amount of water (moisture) taken up by food/powder to achieve a desirable consistency and to create a quality food product [92, 93]. The higher the moisture content of food, the lower the WAC [94, 95]. The WAC for the experimental soup samples was significantly (*p* < 0.05) higher than that of the control sample. Among the F1 variations, the addition of cowpea leaf powder and pumpkin seed powder had a significant effect on the WAC—an inverse effect was noted whereby a reduction in the WAC from 214 to 185.7 g/g was associated with an increase in cowpea leaf powder and pumpkin flower powder. Comparable results were also obtained by Sharma and Lakhawat [96], who also obtained a WAC of 189 g/g in pumpkin seed powder. The results are also aligned with the findings by Kumari et al. [97], who reported a WAC for pumpkin seed flour ranging from 178.00 to 296.10 g/g. The decrease in WAC may be attributed to the low protein content in cowpea leaf powder. Proteins are both hydrophobic and hydrophilic in nature and so can interact with the water in foods [92, 98], and according to Khalid and Elharadallou [99], a low protein content contributes to reduced water absorption.

A similar trend was observed for the variations of F3, where the WAC decreased from 206.3 to 185.7 g/g. However, variations for F2 followed an opposing trend where the WAC increased from 252 to 263.3 g/g. As observed by Oyedeji et al. [95] and Köhn et al. [94], a decrease in the WAC of F3 variations was probably due to the higher moisture content of cowpea seeds and pumpkin fruits. On the other hand, the increasing WAC values for F2 variations may be linked to the higher protein content of cowpea seeds [100]. According to Emeka-Ike and Chukwuemeka [101], a high WAC could be attributed to increased levels of carbohydrates present in food. The increased WAC of F2 could potentially have a functional impact on preventing the separation of liquid from the soup, known as syneresis [102].

In a recent investigation conducted by Shevchenko, Drobot, and Galenko [103], where the utilization of pumpkin seed flour in baked goods was examined, the study revealed that the WAC of pumpkin seed flour was 1.5 times higher than that of wheat flour. The quality of food products can be adversely affected by either exceptionally low or excessive water absorption, as highlighted by Awuchi, Igwe, and Echeta [92]. Therefore, the moderate WAC results obtained in this study play a crucial role in maintaining the consistency of the soup, as emphasized by Iwe, Onyeukwu, and Agiriga [104]. This finding further supports the suitability of cowpea and pumpkin for soup formulations.

#### 3.2.2. SP of Cowpea–Pumpkin Composite Soup Formulations

The SP is the volume in milliliters taken up by the swelling of 1 g of food material under specific conditions [92]. The SP is considered a quality measure in some food products as it is an indication of the noncovalent bonding between molecules of starch granules and is also one of the factors in determining the a-amylose and amylopectin ratios [104]. The results from the current study regarding the determination of SP are shown in Table 4 and indicate that the SP for the experimental samples showed mean values ranging from 349% to 471%, while the control sample showed an intermediate mean value of 437%.

Among the variations of F1, the addition of cowpea leaf powder and pumpkin seed powder significantly increased the (*p* < 0.05) SP of the soup mix compared to the control. This is shown by the upward trend where the SP increased from 349% to 445% with an increase in cowpea leaf powder and pumpkin seed powder. This increase in the SP of F1 was probably due to the high starch and protein content of pumpkin seed [105, 106]. The elevated WAC of pumpkin seed powder is directly correlated with its high SP, which can be attributed to its abundant starch content [105, 106] and the strong affinity of pumpkin seed proteins towards water. A similar trend was also observed for the variations of F3, where the SP increased from 364% to 471% following an increase in cowpea leaf powder and pumpkin flower. Surprisingly, this increase in SP of F3 was not expected, as the constituents of the composite mix were pumpkin flower and cowpea leaf powders. The assumption was that these constituents could have interfered with the interaction between starch and water, resulting in low swelling of the starch [105, 106]. According to Surech and Samsher [107], a high starch content increases the SP (index) of foods and the increase in SP was probably due to the high amounts of starch reported in all the samples.

F2 did not exhibit a distinct trend for SP mean values, as the findings seemed to be a combination of different results. However, F2 exhibited the highest SP mean values compared to F1 and F3 at 5% and 10% levels of composite mix addition. The higher SP of F2 may be attributed to the high starch content of pumpkin fruit [108]. Given the F2 variations in which starch displayed high mean values of SP, it is possible that this starch contributed to their low crystallinity [109].

#### 3.2.3. Dispersibility of Cowpea–Pumpkin Composite Soup Formulations

The ease with which the powder scatters as individual particles in the bulk liquid phase is known as its dispersibility [110]. The capacity of a mixture to disperse in water serves as an indication of its ability to be reconstituted [111]. In this study, the addition of pumpkin seed, fruit, flower, cowpea leaf, and seed powder had a significant (*p* < 0.05) effect on the dispersibility of the formulations, as shown by a decrease of 22.7% when compared to the control (62.7%) (Table 4). Among the variations of F1, the dispersibility increased with the addition of cowpea leaf powder and pumpkin seed powder, as shown by an increase from 57.7% to 61.3%. However, F1 at the 10% level of composite mix addition displayed a dispersibility mean value comparable to that of the control soup, but F2 and F3 had dispersibility mean values that were significantly (*p* < 0.05) lower than the control soup.

Like in the case of SP, F1 and F3 followed an upward trend of dispersibility as the concentration of the composite mix increased. It appears that the composite mix's high dispersibility facilitated its reconstitution; the more dispersible the composite mix was, the more effectively it could be reconstituted in water [110]. Conversely, a downward trend was noted among the soup variations of F2, where dispersibility decreased from 47.0% to 22.7% with the addition of cowpea seed powder and pumpkin fruit powder. A similar trend was also observed in variations of F3, where the dispersibility decreased from 56.7% to 49.3% with an increase in cowpea leaf powder and pumpkin flower powder. The need to improve the dispersibility of our experimental soups, as shown in the study by Ssepuuya, Katongole, and Tumuhimbise [110], is emphasized. Enhancing the dispersibility to a range of 74% to 77% would lead to better reconstitution for soup mixes.

#### 3.2.4. Rehydration Ratio of Cowpea–Pumpkin Composite Soup Formulations

Rehydration is a way of analysing dehydrated items, whereby a substantial rehydration ratio signifies that the dried product exhibits the desired quality of allowing water to permeate through its pores and reenter the cells [112]. In this study, the addition of cowpea and pumpkin powder led to a significant (*p* < 0.05) increase in the rehydration ratio compared to the control soup mix (Table 4). The rehydration ratio of F2 was significantly (*p* < 0.05) higher than that of F1, F3, and the control. F2 variations significantly (*p* < 0.05) differed from the control, while F1 at all concentrations of composite mix and F3 at 5% and 10% of composite mix did not significantly differ from the control. All the formulations followed an upward trend as the concentration of the composite mix increased.

Among the variations of F1, the rehydration ratio increased from 8.0 to 8.7 g/g with increasing concentrations of cowpea leaf and pumpkin seed powder. Pumpkin seeds contain high protein and starch content; therefore, higher protein content may contribute to improved water absorption and increased hydration in soup mixes [33, 105, 106], in addition to the remarkable hydration properties of starch [113, 114]. The variation of F3 had a rehydration ratio increasing from 8.0 to 10.7 g/g as the concentration of cowpea leaf powder and pumpkin flower increased. This increase in rehydration ratio may be attributed to the presence of fibre in the formulations, as fibre is known to have a high WAC which could have in turn influenced the hydration of the soup mix [115]. Among the variations of F2, the rehydration ratio increased from 12.3 to 14.7 g/g with an increase in cowpea seed powder and pumpkin fruit. The increase in rehydration ratio may be attributed to the high content of starch present in the samples. Products that exhibit a heightened capacity for rehydrating are tastier and preserve their visual appeal [116]. The high rehydration ratios obtained from this study indicate that cowpea and pumpkin are suitable for soup production. A high rehydration ratio means that the soup mix will absorb a lot of water, resulting in a thick and hearty soup. This is ideal for soups that are meant to be filling and hearty, such as stews, chowders, and bisques. On the other hand, a low rehydration ratio produces a thinner soup, which is better suited for light and brothy soups like consommés and clear broth soups. Having a soup mix with a high rehydration ratio allows for more flexibility in the soup-making process.

### 3.3. Effect of Cowpea Powder and Pumpkin Powder on TPC and AA of Cowpea–Pumpkin Composite Soup

The TPC of the cowpea–pumpkin composite soup samples are presented in Table 5. The TPC of F1 showed significantly (*p* < 0.05) lower mean values (0.25–0.32 mg GAE/100 g) than the control (0.44 mg GAE/100 g), F2 with 15% composite mix (0.54 mg GAE/100 g), and F3 at all concentrations of composite mix (0.54–0.63 mg GAE/100 g). The TPC of F3 (0.54–0.63 mg GAE/100 g) was significantly (*p* < 0.05) higher than that of F1 and F2, including the control (0.44 mg GAE/100 g). The TPC of F1 decreased as the concentration of the composite mix increased, while an opposite trend was observed for F2 and F3. Interestingly, it is assumed that the leaves and seeds could increase the TPC of the soup as they contain high amounts of phenolic compounds [21, 117–119]. However, the TPC of F1 and F2 seems to have been affected by the presence of cowpea and pumpkin seeds which are known to be substantially high in dietary proteins that coexist with polyphenolic compounds [120–122]. The interaction between protein and phenolic compounds probably resulted in the formation of complexes or aggregates which probably entrapped the phenolic compounds and therefore prevented their release into the soup matrix [123].

TPC of F3 increased with increased concentrations of cowpea leaf and pumpkin flower powders, and this was probably due to the individual contribution of phenolic compounds naturally found in cowpea leaf and pumpkin flower [124–126]. Tzanova et al. [20] conducted a study on the antioxidant potential of different genotypes of cowpeas, and their findings align with the results obtained in this study. They discovered that cowpea leaves have a significant amount of TPC. Similarly, Santos et al. [30] conducted a separate study and observed that incorporating pumpkin flower into the patty formulation led to an increase in phenolic content.

The concentration of composite mixes had a direct impact on the AA of F1 and F2, resulting in a decrease. In the case of F1, this decrease in AA was evident in the ABTS RSA, which decreased from 57.01 to 32.94 *μ*M TE/g sample, and the DPPH RSA, which decreased from 16.40% to 16.24%. This decrease corresponded with the decrease in TPC, which decreased from 0.32 to 0.25 mg GAE/100 g. On the other hand, F2 exhibited mixed responses, as the AA decreased from 61.74 to 48.32 *μ*M TE/g sample (ABTS) and DPPH RSA from 15.91 to 15.88%, while the TPC increased from 0.32 to 0.54 mg GAE/100 g.

The reasons for the antagonistic reactions between TPC and AA in F2 remain unclear, but it is possible that phenolic compounds were masked due to phenol–protein complexation. In contrast, F3 showed a significant increase in TPC, ranging from 0.54 to 0.63 mg GAE/100 g, as well as an increase in AA from 66.47 to 72.40 *μ*M TE/g sample (ABTS) and DPPH RSA from 16.22% to 16.31%. This observation supports the speculation that the presence of protein-rich ingredients had a negative effect on the extractability of phenolic compounds in F1 and F2, as F3, which did not contain any protein-rich ingredients, exhibited higher TPC and AA. The control had significantly (*p* < 0.05) higher ABTS RSA (77.06 *μ*M TE/g sample) compared to the cowpea–pumpkin composite soup formulations (F1, F2, and F3), while the DPPH RSA of the control was significantly (*p* < 0.05) lower than that of F1 and F3. The high TPC and AA of F3 can be attributed to the fact that the phenolic compounds of cowpea leaf powder and pumpkin flower powder did not interact with potential chelating constituents such as dietary proteins.

### 3.4. Effect of Cowpea Powder and Pumpkin Powder on Consumer Acceptability of Cowpea–Pumpkin Composite Soup

**Figure 2:**
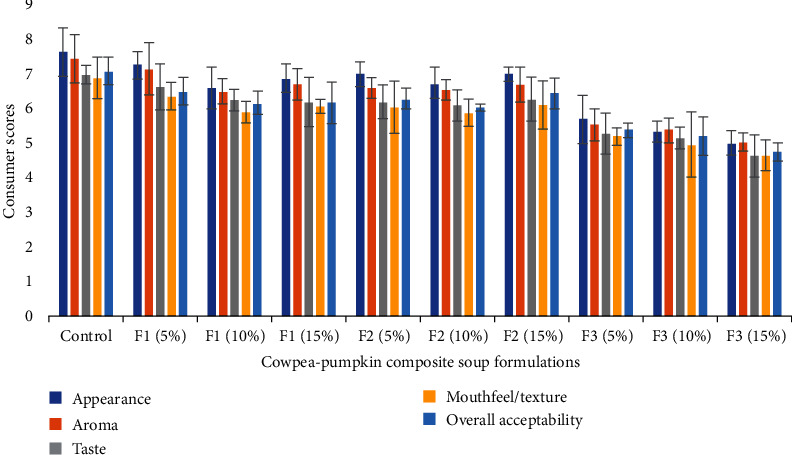
Consumer acceptability of cowpea–pumpkin composite soup samples.

Consumer acceptance of the soup sample result is presented in Figure 2. In general, the experimental soups were less acceptable than the control. The results indicate that the consumer acceptance scores on the appearance of F1 and F2 did not significantly differ from the control sample, despite the slightly lower mean values. This similarity in the appearance of F1 and F2 samples can be attributed to their attractive colour, a result of incorporating pumpkin seeds in F1 and pumpkin fruit in F2, highlighting the vital role of colour in food acceptance. Similarly, the influence of colour on consumer preferences was reported in a study by Arifin, Siti Nur Izyan, and Huda-Faujan [76], which reported the sensory appeal of muffins made with pumpkin puree.

Contrary to the visual examination of the soups, F2 (15%) with pumpkin fruit and cowpea seed scored the highest acceptance on appearance. The F2 acceptance score on appearance was similar to F1 with 5% composite mix where cowpea leaf powder and pumpkin seed powder were added. The use of pumpkin as a food ingredient has consistently demonstrated its superiority in terms of visual appeal, and the incorporation of pumpkin powder into these formulations has yielded desirable effects [76, 127–130]. The addition of pumpkin seeds to the F1 formulation could have neutralized the less acceptable colour of cowpea leaves. The appearance of F3 at all levels of composite mix incorporation was significantly (*p* < 0.05) less acceptable compared to that of the control. The lower acceptability score on the appearance of F3 can be attributed to the presence of cowpea leaf powder, resulting in a dark green soup colour that is less appealing to the consumer panel visually.

The control exhibited the highest aroma mean score, which did not differ significantly from that of F1 and F2, with mean values ranging from 6.51 to 7.14. The addition of pumpkin seed powder to F1 samples contributed to the acceptable aroma. The same results are also observed for F2 samples in this study, where a combination of cowpea seeds and pumpkin fruit produced a good aroma.

Biggins, Qian Oo, and Weaver [131] reported high consumer scores for the aroma of cakes made with ground pumpkin seed meal, while Moongngarm et al. [132] found cowpea seed powder cookies acceptable for odour and overall liking. The F3 samples received the lowest aroma scores, and the low mean scores resulted from the strong odour of cowpea leaves which was probably deemed highly unacceptable by consumers. This could be linked to the presence of sulfide volatile compounds in cowpea leaves, although in small quantities, that can cause an unpleasant and undesirable odour [133].

The results show that consumer acceptance of soup taste for F1, F2, and control samples was similar, as shown by their acceptance scores ranging from 6.10 to 6.99. The rich, nutty taste of pumpkin seed powder used in formulating F1 samples could be the reason for consumer acceptance. According to the findings reported by Jeong-Yeon et al. [134], pumpkin seeds possess a distinct taste and flavour. The taste of the F2 samples was found to be equally well accepted as the F1 samples. This is attributable to the inclusion of cowpea seed and pumpkin fruit powders in the soup formulations. A study by Borro and Gemora [127] reported that pumpkin fruit added to cake tasted savoury and was very acceptable. It was also reported that pumpkin fruit is sweet [135] and can be used in many mixes because of its attractive flavour, colour, and sweetness [76, 136, 137].

The F3 containing 5%, 10%, and 15% composite mixes were observed to have the lowest acceptance scores for taste. This finding indicates that an increase in the concentration of pumpkin flower and cowpea leaf powder resulted in an unacceptable soup taste. This undesirable taste could be associated with the tannins in cowpea leaf powder [21, 138]. The control soup was highly rated for mouthfeel/texture. F1 and F2 at the increasing mix concentration levels did not significantly vary from the control. It is noteworthy that food items made from pumpkin fruit and seed powder were more widely accepted in terms of their texture and acceptability [137]. The lowest acceptance scores were recorded for F3 samples. In the present study, the mouthfeel/texture score of soup decreased with the increasing incorporation of cowpea leaf powder and pumpkin flower powders in the formulations of the soups, specifically the cowpea leaf powder.

The overall results indicate that the acceptance of the control soup was higher than the experimental formulations but did not significantly differ from that of the F1 and the F2. The F3 samples exhibited the lowest overall acceptance mean values ranging from 4.76 to 5.39. The findings also showed that the overall acceptance of soups decreased with the increased inclusion of cowpea leaf and pumpkin flower. In general, the overall acceptability of soups depends on the individual data for sensory attributes such as appearance, aroma, taste, and mouthfeel. The ratings for soup samples made from pumpkin fruit powder and pumpkin seeds and those made from cowpea seed powder and pumpkin fruit powder were found in the range of “like extremely” to “like very much.”

### 3.5. Correlation Between Functional Properties, Viscosity, and Consumer Acceptability of Cowpea–Pumpkin Composite Soup Formulations

A positive correlation was observed among the functional properties of the soups, with RR significantly correlating with WAC (0.675) and SP (0.616) (Table 6). This suggests that the high WAC of the cowpea–pumpkin soup formulations improved their rehydration properties, which in turn influenced the SP of these formulations. The dispersibility of the formulations was found to positively correlate with sensory acceptability. Moreover, a positive correlation was observed between viscosity and WAC, indicating that as the starchy and proteinaceous solids absorbed more water, the soup became more viscous.

The higher viscosity of the soup was associated with the WAC of the solids present in the formulation. However, the increase in viscosity negatively affected the sensory attributes of the soups, as indicated by the negative correlation between viscosity and their acceptability. Furthermore, a strong positive correlation was found among all the sensory attributes.

## 4. Conclusion

The objective of this study was to develop a cost-effective soup formulation using ingredients that are readily available from a household garden and suitable for use in the kitchens of families with limited financial resources. F2 was found to be low in TPC and AA in comparison to F3 but still exhibited the potential for further advancement and commercialization due to its positive consumer feedback and favourable sensory characteristics. In general, F2, which consisted of 10% cowpea seed powder and pumpkin fruit powder, appeared to be an ideal soup blend. It resulted in an appealing golden brown soup with enhanced SP and viscosity, which could aid in swallowing. The findings of this study hold significant importance for low-income households as they offer the potential to facilitate the preparation of healthier culinary alternatives that are well-received by consumers.

## Figures and Tables

**Table 1:** Formulation of soup mixes.

**Ingredients**	**Control soup**	**F1 (5%)**	**F1 (10%)**	**F1 (15%)**	**F2 (5%)**	**F2 (10%)**	**F2 (15%)**	**F3 (5%)**	**F3 (10%)**	**F3 (15%)**
Dry carrots	16.20	16.20	16.20	16.20	16.20	16.20	16.20	16.20	16.20	16.20
Potato powder	19.03	19.03	19.03	19.03	19.03	19.03	19.03	19.03	19.03	19.03
Onion powder	4.05	4.05	4.05	4.05	4.05	4.05	4.05	4.05	4.05	4.05
Mushroom powder	5.67	5.67	5.67	5.67	5.67	5.67	5.67	5.67	5.67	5.67
Powdered maltodextrin	24.29	24.29	24.29	24.29	24.29	24.29	24.29	24.29	24.29	24.29
Hydrogenated fat	12.15	12.15	12.15	12.15	12.15	12.15	12.15	12.15	12.15	12.15
Salt	4.86	4.86	4.86	4.86	4.86	4.86	4.86	4.86	4.86	4.86
Brown sugar	3.24	3.24	3.24	3.24	3.24	3.24	3.24	3.24	3.24	3.24
Tomato powder	10.53	10.53	10.53	10.53	10.53	10.53	10.53	10.53	10.53	10.53
Composite mix A	—	5	10	15	—	—	—	—	—	—
Composite mix B	—	—	—	—	5	10	15	—	—	—
Composite mix C		—	—	—	—	—	—	5	10	15
Total (g)	100	105	110	115	105	110	115	105	110	115

*Note:* Control contain 0% composite mixes. Using the control soup as a base, the composition of the soup mix formulations varied by the addition of components at 5%, 10, or 15% (based on 100 g composite mix) as follows: Formulation 1: increasing proportions of powdered cowpea leaf and pumpkin seed. Formulation 2: increasing proportions of powdered cowpea seed and pumpkin fruit. Formulation 3: increasing proportions of powdered cowpea leaf and pumpkin flower.

**Table 2:** Colour of cowpea–pumpkin composite soup formulations.

**Soup formulations**		**L** ^∗^	**a** ^∗^	**b** ^∗^	**C** ^∗^
Control		71.96 ± 0.01^h^	4.19 ± 0.02^i^	16.50 ± 0.01^c^	17.02 ± 0.01^d^
Formulation 1	5%	74.06 ± 0.02^j^	−3.20 ± 0.02^f^	19.83 ± 0.01^g^	20.09 ± 0.01^g^
	10%	67.35 ± 0.01^f^	−0.43 ± 0.02^b^	15.13 ± 0.01^a^	15.14 ± 0.01^a^
	15%	60.92 ± 0.01^e^	−0.63 ± 0.02^d^	16.48 ± 0.02^c^	16.49 ± 0.02^b^

Formulation 2	5%	72.49 ± 0.01^i^	3.72 ± 0.01^g^	23.55 ± 0.01^h^	23.84 ± 0.01^h^
	10%	58.24 ± 0.01^c^	0.50 ± 0.02^c^	17.41 ± 0.01^d^	17.42 ± 0.01^e^
	15%	55.29 ± 0.00^b^	0.96 ± 0.02^e^	17.51 ± 0.02^e^	17.54 ± 0.02^f^

Formulation 3	5%	71.37 ± 0.02^g^	3.81 ± 0.05^h^	26.86 ± 0.01^i^	27.13 ± 0.01^i^
	10%	59.07 ± 0.01^d^	3.02 ± 0.01^a^	17.56 ± 0.01^f^	17.56 ± 0.01^f^
	15%	52.63 ± 0.01^a^	3.20 ± 0.01^f^	16.29 ± 0.02^b^	16.60 ± 0.02^c^

*Note:*Mean ± standard deviation. Mean values followed by different letters in a column are significantly different (*p* < 0.05) (LSD). *L*^∗^ denotes lightness from black (0) to white (100), *a*^∗^ denotes redness (+) to greenness (−), and *b*^∗^ denotes yellowness (+) to blueness (−) [75]. The chroma (*C*^∗^) coordinate indicates colour intensity [4].

**Table 3:** Rheological properties of cowpea–pumpkin soup formulations at 26°C.

**Soup formulations**		**Viscosity (cP)**	**Shear stress (Pa)**	**Shear rate (s** ^**−1**^ **)**
Control		33.21 ± 0.04^c^	13.92 ± 0.19^c^	497.23 ± 2.05^a^
Formulation 1	5%	88.91 ± 1.10^i^	21.21 ± 0.30^f^	497.23 ± 0.85^a^
	10%	31.92 ± 0.13^c^	36.38 ± 0.19^h^	752.56 ± 0.41^c^
	15%	56.64 ± 0.45^e^	15.14 ± 0.55^d^	497.23 ± 0.95^a^

Formulation 2	5%	21.09 ± 0.40^a^	7.80 ± 0.10^a^	497.20 ± 0.30^a^
	10%	62.75 ± 0.16^f^	38.55 ± 0.11^i^	653.24 ± 0.29^b^
	15%	48.78 ± 0.85^d^	18.96 ± 0.18^e^	497.11 ± 2.70^a^

Formulation 3	5%	24.13 ± 0.55^b^	8.65 ± 0.05^b^	497.20 ± 0.50^a^
	10%	80.67 ± 0.25^h^	65.13 ± 0.04^j^	823.82 ± 0.14^d^
	15%	71.50 ± 0.70^g^	22.37 ± 0.56^g^	497.21 ± 1.39^a^

*Note:*Mean ± standard deviation. Mean values followed by different letters in a column are significantly different (*p* < 0.05) (LSD).

**Table 4:** Functional properties of cowpea–pumpkin composite soup formulations.

**Soup formulations**		**WAC (g/g)**	**SP (%)**	**Dispersibility (%)**	**RR (g/g)**
Control		65.7 ± 1.5^a^	437.0 ± 2.0^e^	62.7 ± 1.5^e^	7.7 ± 0.6^a^
Formulation 1	5%	214.0 ± 1.7^e^	349.0 ± 2.0^a^	57.7 ± 2.1^d^	8.0 ± 1.0^ab^
	10%	185.7 ± 1.5^b^	354.0 ± 2.0^b^	61.3 ± 2.1^de^	8.7 ± 0.6^abc^
	15%	194.3 ± 1.5^c^	445.0 ± 2.0^f^	59.0 ± 1.0^de^	8.7 ± 0.6^abc^

Formulation 2	5%	252.0 ± 1.0^f^	464.0 ± 1.7^h^	47.0 ± 2.0^c^	12.3 ± 1.5^de^
	10%	252.0 ± 2.0^f^	445.3 ± 1.5^f^	39.3 ± 1.5^b^	11.3 ± 0.6^cd^
	15%	263.3 ± 2.0^g^	451.7 ± 1.2^g^	22.7 ± 2.1^a^	14.7 ± 1.5^e^

Formulation 3	5%	206.3 ± 1.5^d^	364.7 ± 1.5^c^	56.7 ± 1.5^d^	8.0 ± 1.0^ab^
	10%	185.7 ± 1.5^b^	423.3 ± 1.5^d^	51.7 ± 1.5^c^	10.0 ± 1.0^abcd^
	15%	189.3 ± 1.5^b^	471.0 ± 1.0^i^	49.3 ± 1.5^c^	10.7 ± 0.6^bcd^

*Note:*Mean ± standard deviation. Mean values followed by different letters in a column are significantly different (*p* < 0.05) (LSD).

Abbreviations: RR, rehydration ratio; SI, solubility index; SP, swelling power; WAC, water absorption capacity.

**Table 5:** Total phenolic content and antioxidant activity of cowpea–pumpkin composite soup formulations.

**Soup formulations**		**TPC (mg GAE/100 g)**	**ABTS (** ***μ*** **M TE/g sample)**	**DPPH radical scavenging activity (%)**
Control		0.44 ± 0.01^c^	77.06 ± 3.75^h^	15.97 ± 0.03^b^
Formulation 1	5%	0.32 ± 0.01^b^	57.01 ± 3.43^d^	16.40 ± 0.04^f^
	10%	0.30 ± 0.01^b^	41.12 ± 1.71^b^	16.24 ± 0.03^cd^
	15%	0.25 ± 0.01^a^	32.94 ± 1.03^a^	16.33 ± 0.04^ef^

Formulation 2	5%	0.32 ± 0.01^b^	61.74 ± 1.92^de^	15.91 ± 0.01^ab^
	10%	0.47 ± 0.02^c^	48.96 ± 0.61^c^	15.90 ± 0.02^ab^
	15%	0.54 ± 0.03^d^	48.32 ± 1.35^c^	15.88 ± 0.01^a^

Formulation 3	5%	0.54 ± 0.01^d^	66.47 ± 1.78^ef^	16.22 ± 0.02^c^
	10%	0.63 ± 0.02^e^	72.40 ± 0.71^gh^	16.31 ± 0.07^de^
	15%	0.63 ± 0.01^e^	68.58 ± 0.49^fg^	16.29 ± 0.04^cde^

*Note:*Mean ± standard deviation. Mean values followed by different letters in a column are significantly different (*p* < 0.05) (LSD).

Abbreviations: ABTS, 2,2′-azino-bis-3-ethylbenzothiazoline-6-sulfonic acid antioxidant assay; DPPH, 2,2-diphenyl-l-picrylhydrazyl antioxidant assay; TPC, total phenolic content.

**Table 6:** Correlation between functional properties, viscosity, and consumer acceptability of cowpea–pumpkin composite soup formulations.

**Variables**	**RR**	**WAC**	**SP**	**Disp**	**Viscosity**	**App**	**Aroma**	**Taste**	**M/T**	**OA**
RR	1	0.675	0.616	−0.940	−0.027	−0.024	−0.102	−0.123	−0.122	−0.059
WAC	0.675	1	0.093	−0.688	0.104	−0.111	−0.186	−0.203	−0.254	−0.221
SP	0.616	0.093	1	−0.493	−0.006	−0.071	−0.117	−0.173	−0.097	−0.099
Disp	−0.940	−0.688	−0.493	1	−0.085	0.009	0.076	0.100	0.101	0.043
Viscosity	−0.027	0.104	−0.006	−0.085	1	−0.253	−0.178	−0.229	−0.261	−0.295
App	−0.024	−0.111	−0.071	0.009	−0.253	1	0.992	0.987	0.989	0.986
Aroma	−0.102	−0.186	−0.117	0.076	−0.178	0.992	1	0.994	0.993	0.985
Taste	−0.123	−0.203	−0.173	0.100	−0.229	0.987	0.994	1	0.991	0.990
M/T	−0.122	−0.254	−0.097	0.101	−0.261	0.989	0.993	0.991	1	0.995
OA	−0.059	−0.221	−0.099	0.043	−0.295	0.986	0.985	0.990	0.995	1

*Note:* Correlation significant at 0.05 level.

Abbreviations: App, appearance; Disp, dispersibility; M/T, mouthfeel/texture; OA, overall acceptability; RR, rehydration ratio; SP, swelling power; WAC, water absorption capacity.

## Data Availability

The data used to substantiate the findings of this study are contained in the article. Nevertheless, additional information required can be obtained from the corresponding author upon request.
