# Correction to “Effect of Cowpea and Pumpkin Powders on the Physicofunctional Properties, Total Phenolic Content, Antioxidant Activity, and Consumer Acceptability of Soup”

**DOI:** 10.1155/ijfo/9792486

**Published:** 2025-11-20

N. Mungofa and D. Beswa, “Effect of Cowpea and Pumpkin Powders on the Physicofunctional Properties, Total Phenolic Content, Antioxidant Activity, and Consumer Acceptability of Soup,” *International Journal of Food Science* 2024 (2024): 3596783, 10.1155/2024/3596783

In the article, key details were omitted from Figure 1. The corrected Figure 1 is shown below:

**Figure 1:**
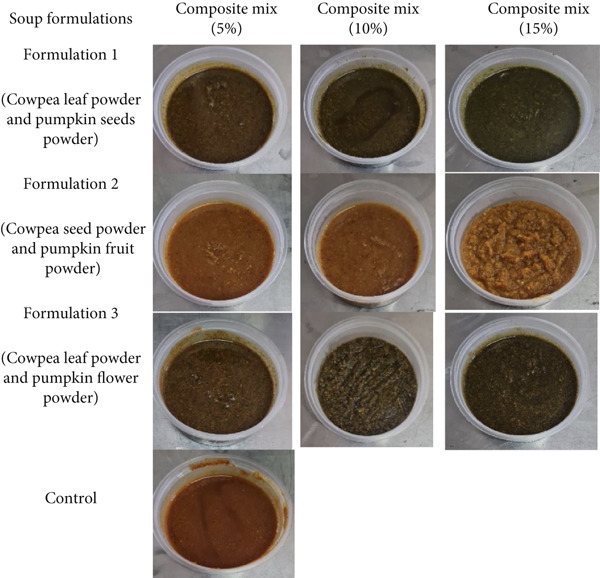
Photographs showing variations in the colour of cowpea–pumpkin composite soup formulations (photographs taken by N.M.).

We apologize for this error.

